# Supplementary material for: Recovering false negatives in CRISPR fitness screens with JLOE
Source: Nucleic Acids Res. 2023 Feb 2;51(4):1637–51. doi: 10.1093/nar/gkad046 (PMC9976895; doi:10.1093/nar/gkad046)
Supplement: gkad046_Supplemental_Files [file gkad046_supplemental_files.zip › Supplementary_Figure_Legends.pdf]

Supplemental Figure 1. A) *the distribution of the F-measures of 769 Avana Screens. The red dashed line indicates F-measure of 0.8 and screens with F-measure >0.8 were considered as high-performing and were retained for downstream analyses.* ***B)*** *Precision-recall curve specifying an example of a good performing screen (MIAPACA2, in blue) and a bad performing screen (WM115, in green). For all Avana screens, precision-recall curves were calculated using the reference gold standard sets of essential and non-essential genes and the point on the precision-recall curve for each screen where the BF crossed 5 (red points) were identified and the F-measure of each screen was calculated at that point.* ***C)*** *Bayes factor of 10 represents a strict threshold corresponding to a posterior probability of gene essentiality of ~99%* ***D)*** *The distribution of the root mean squared deviation (RMSE) values for each simulation reveals a range of models with RMSE < 2xRMSE$_{min}$ indicated by the red dashed line.*

Supplemental Figure 2. *The cumulative essentials curves for lineages represented by more than or equal to 16 high-quality screens. Sets of 16 cell lines were randomly selected without replacement from all screens and the number of cumulative essential genes with BF>=10 in each consecutive screen were plotted with circles with the error bars indicating the standard deviation of cumulative essential gene observations across 100 iterations. The number of newly discovered essential genes in each consecutive screen were also plotted with triangles. The colors indicate different tissue types labelled in the legend.*

Supplemental Figure 3. *Synthetic genome modeling, applied to each tissue type, estimates the number of essential genes and false discovery rate (FDR) per tissue. Heatmaps showing the root mean squared deviation (RMSE for the models versus the FDR and the number of essential genes in each simulation. The white boxes indicate models with RMSE <2 x RMSE$_{min}$.*

Supplemental Figure 4. *The best fitting models from the synthetic genome modeling approach for individual tissue types. The cumulative essentials curves were plotted for the best fitting model indicated by the blue lines and their fit to the Avana data in their corresponding tissue types (cumulative essential genes across sets of 8 call lines randomly selected without replacement from all available screens in that tissue type for 100 iterations) is shown in red.*

Supplemental Figure 5. *The number of genes in each bin and the mean mRNA expression (TPM) of the genes (indicated by the secondary Y-axis in orange) in corresponding bins for each tissue type for putative true positives (TPs) and false positives (FPs). Error bars indicate the standard deviation of expression of genes in each bin.*

Supplemental Figure 6. *Venn diagrams showing the overlap of the number of new common essentials with other core essential gene sets previously defined in* CoRe AdaM (44) and CENtools (45) approaches.
