# Supplementary material for: Recovering false negatives in CRISPR fitness screens with JLOE
Source: Nucleic Acids Res. 2023 Feb 2;51(4):1637–51. doi: 10.1093/nar/gkad046 (PMC9976895; doi:10.1093/nar/gkad046)
Supplement: gkad046_Supplemental_Files [file gkad046_supplemental_files.zip › Supplementary_Table_Legends.pdf]

Supplementary Table Legends

Supplementary Table 1. Bayes Factors for the 659 cell lines used in this study with F-measure above 0.80 post CrisprCleanR processing.

Supplementary Table 2. Table of binwise false discovery rates across 100 iterations for the tissue types investigated in this study.

Supplementary Table 3. Gene frequency observations out of 8 screens across 100 iterations by tissue type.

Supplementary Table 4. Table of 992 common essential genes and high confidence context essential genes in each tissue type.

Supplementary Table 5. Common essential and core essential genes unique to each approach among previously defined core essential genes.

Supplementary Table 6. Table of normalized binary essentiality calls from JLOE.

Supplementary Table 7. Table of normalized joint posterior Log Odds from JLOE.
