# Supplementary figures and images for: Recovering false negatives in CRISPR fitness screens with JLOE
Source: Nucleic Acids Res. 2023 Feb 2;51(4):1637–51. doi: 10.1093/nar/gkad046 (PMC9976895; doi:10.1093/nar/gkad046)

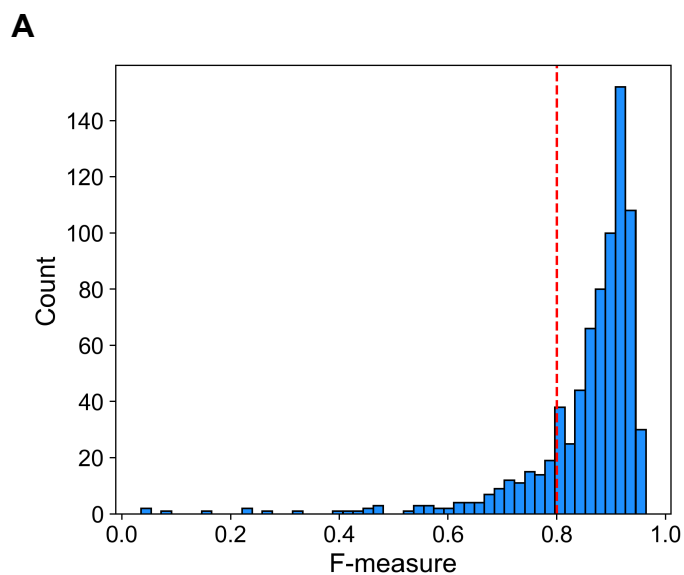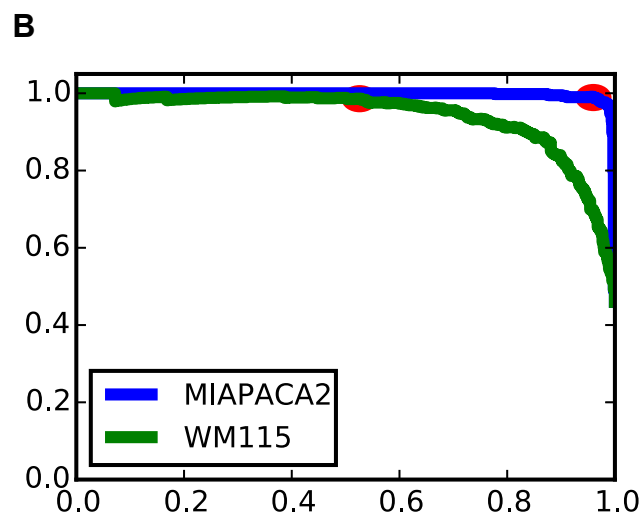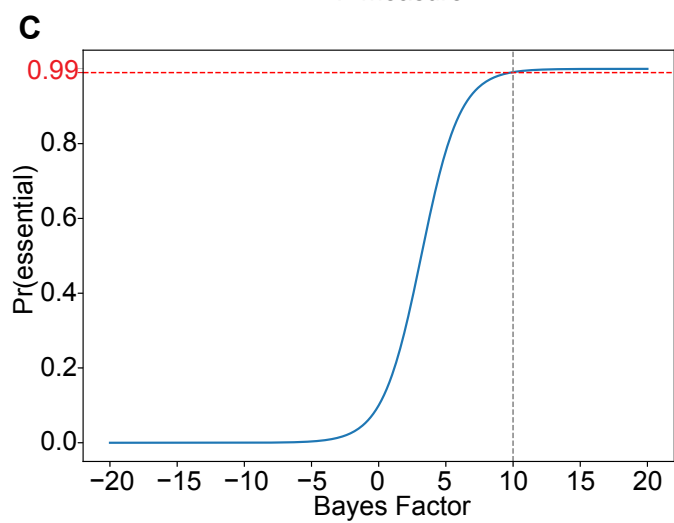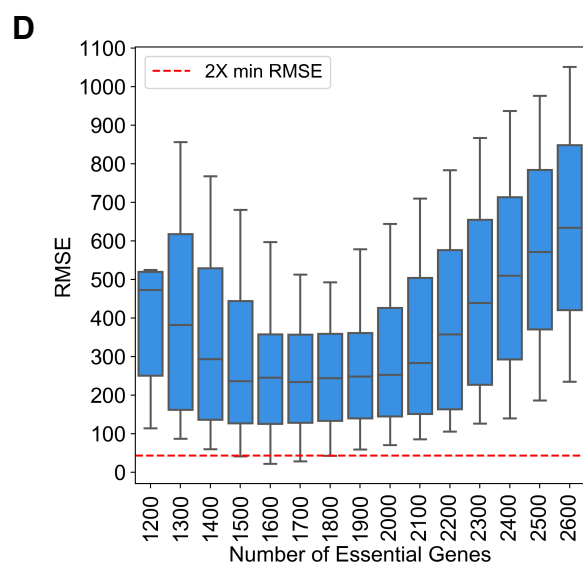

**Supplementary Figure 1.**

Supplement: gkad046_Supplemental_Files [file gkad046_supplemental_files.zip › Supp_Fig1.pdf]

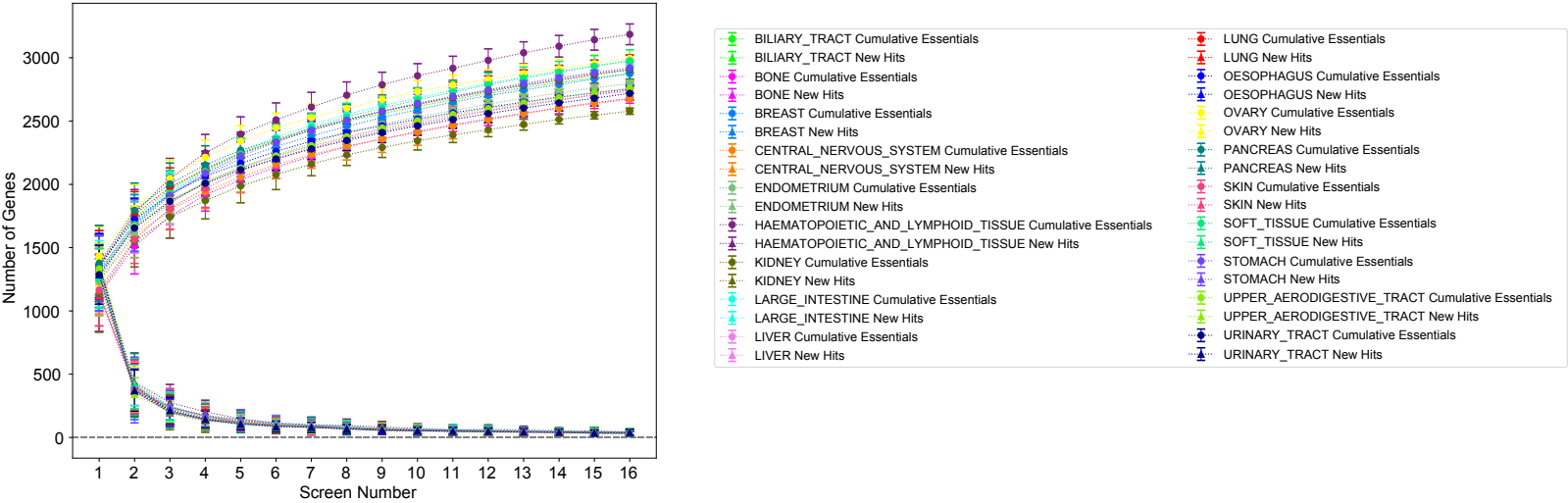

Supplementary Figure 2

Supplement: gkad046_Supplemental_Files [file gkad046_supplemental_files.zip › Supp_Fig2.pdf]

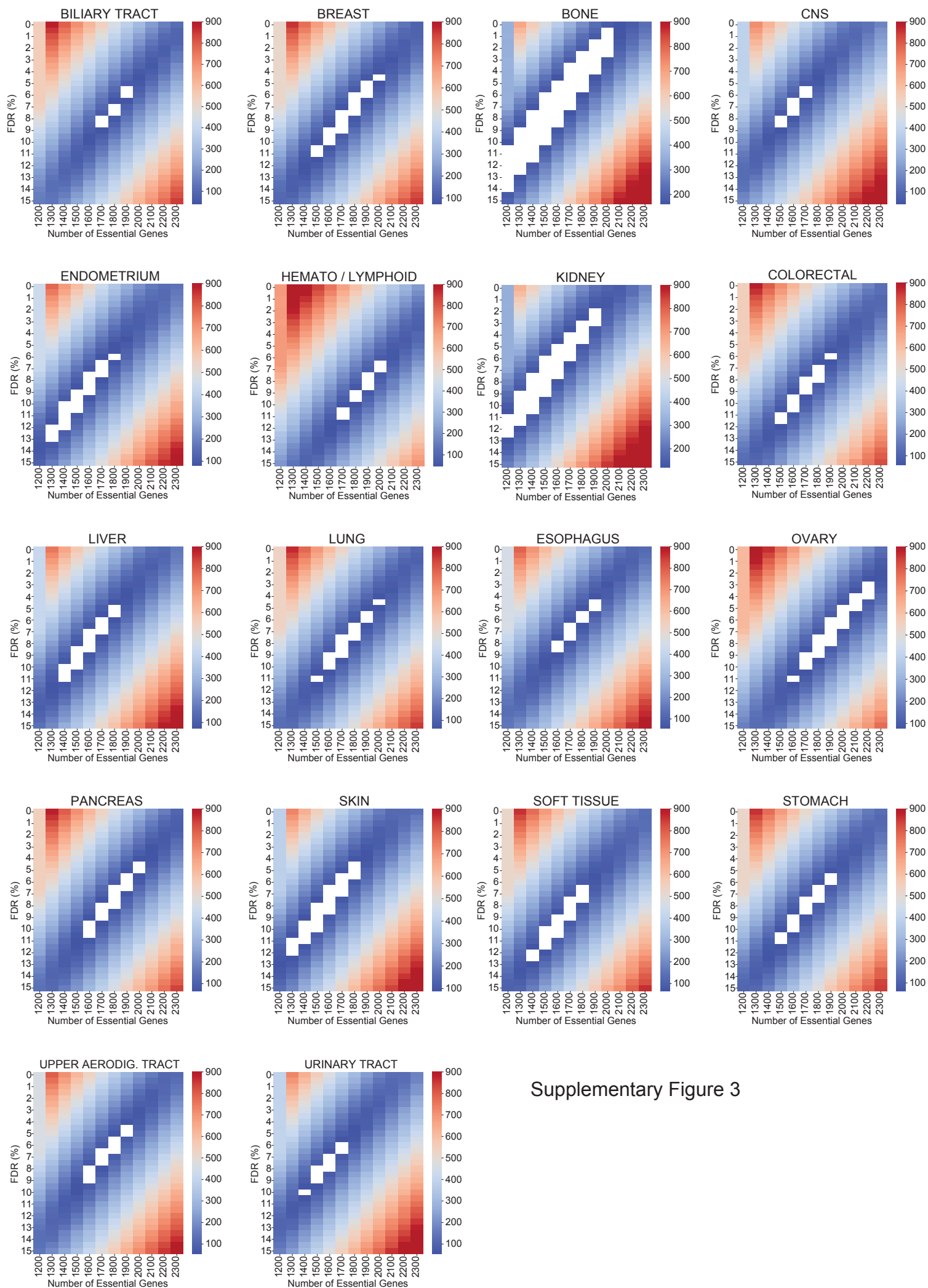

Supplementary Figure 3

Supplement: gkad046_Supplemental_Files [file gkad046_supplemental_files.zip › Supp_Fig3.pdf]

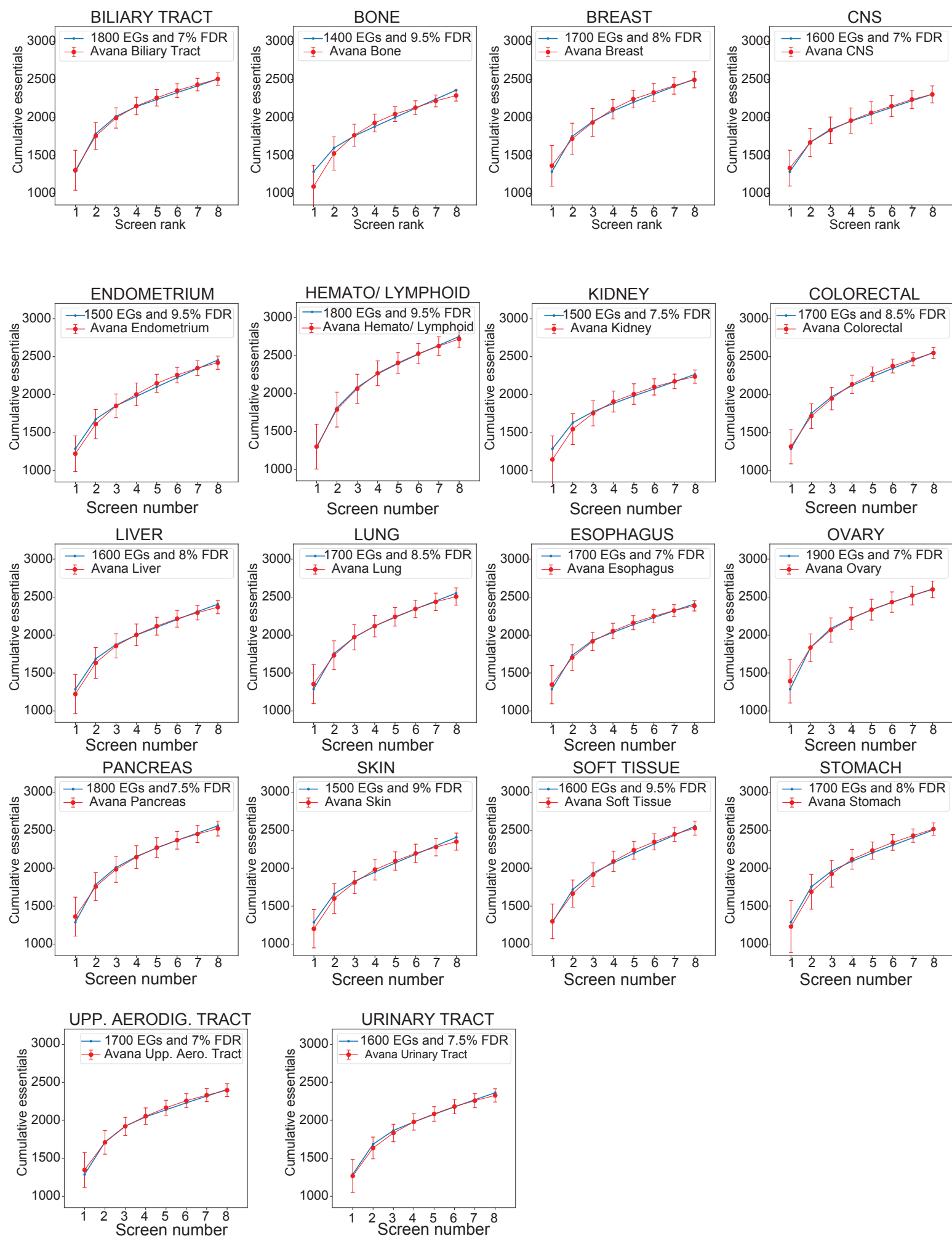

**Supplementary Figure 4.**

Supplement: gkad046_Supplemental_Files [file gkad046_supplemental_files.zip › Supp_Fig4.pdf]

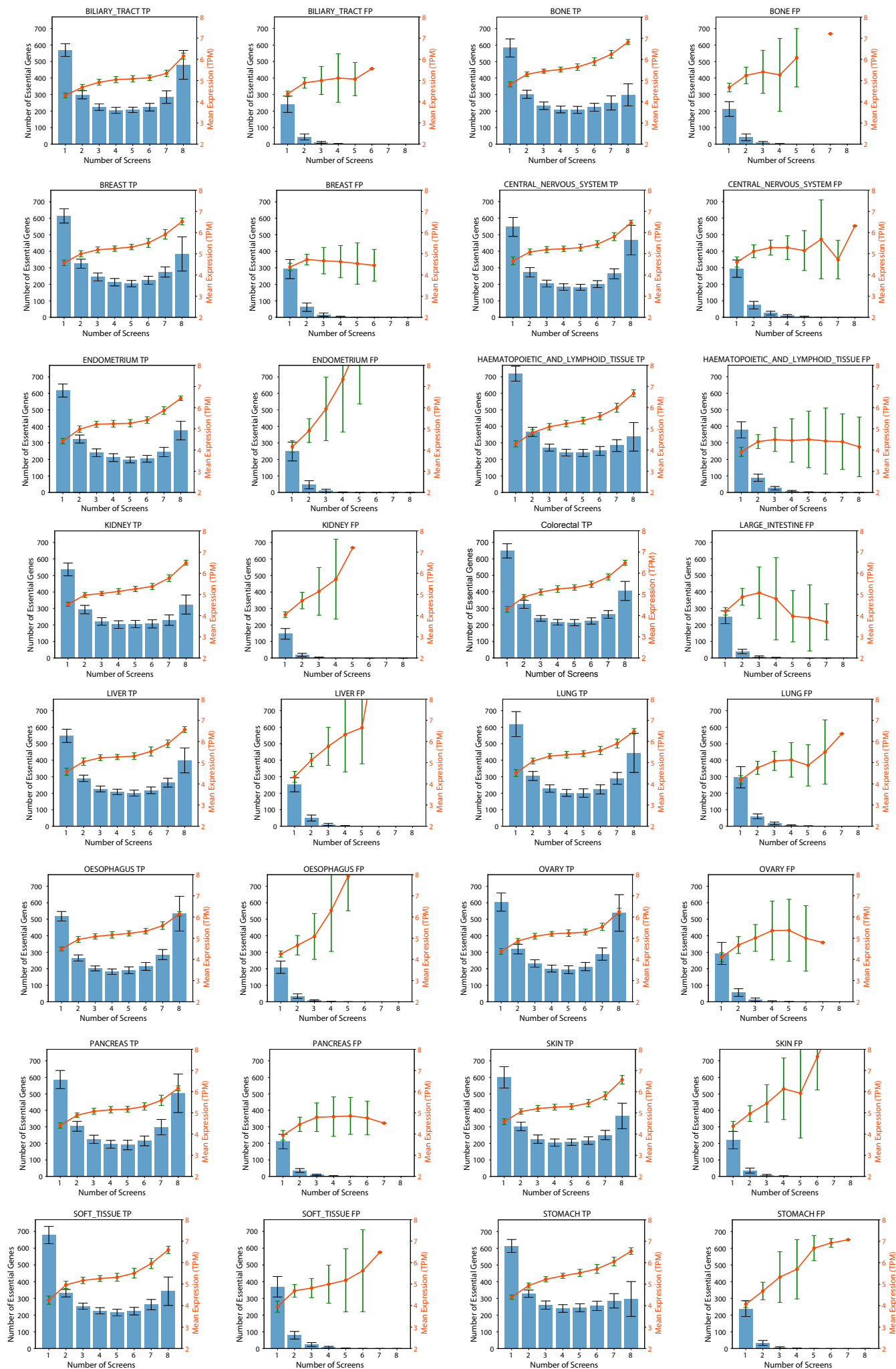

Supplementary Figure 5

Supplement: gkad046_Supplemental_Files [file gkad046_supplemental_files.zip › Supp_Fig5.pdf]

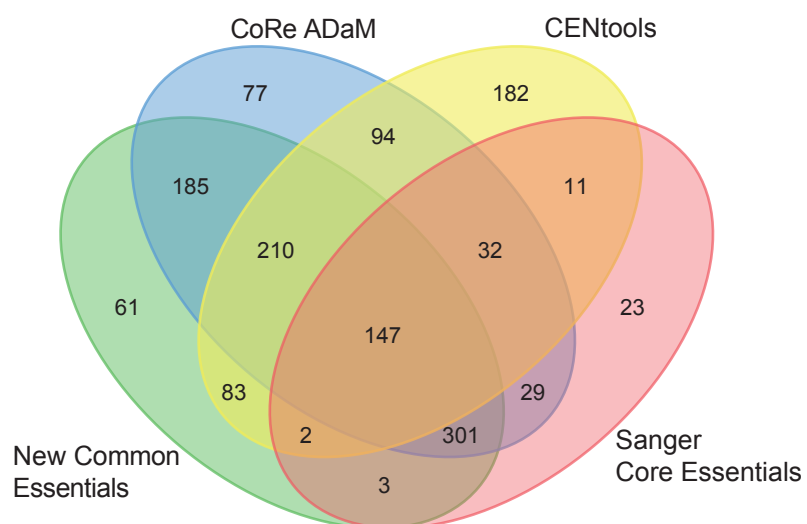

**Supplementary Figure 6**

Supplement: gkad046_Supplemental_Files [file gkad046_supplemental_files.zip › Supp_Fig6.pdf]
